# Effect of Major Illness Insurance on Vulnerability to Poverty: Evidence From China

**DOI:** 10.3389/fpubh.2021.791817

**Published:** 2021-12-23

**Authors:** Lili Zheng, Lijin Peng

**Affiliations:** ^1^School of Insurance, Central University of Finance and Economics, Beijing, China; ^2^Business and Tourism School, Sichuan Agricultural University, Chengdu, China

**Keywords:** major illness insurance, vulnerability to poverty, random forest algorithm, health risk, poverty reduction effect, quasinatural experiment

## Abstract

Disease is the primary cause of poverty in China. Health insurance is an essential mechanism for managing health risks and addressing the risk of financial loss. Using data from the China Family Panel Studies (CFPS) waves from 2010 to 2016, this study develops a random forest method to assess households' vulnerability to poverty and then examines the impact of major illness insurance on vulnerability to poverty by focusing on the rollout period of a major illness insurance scheme. The research also examines the impact of increased major illness insurance coverage on poverty reduction by focusing on the change from low- to high-coverage health insurance. The findings indicate that major illness insurance and improvements in the degree of coverage significantly reduce vulnerability to poverty. In addition, major illness insurance is found to alleviate the vicious cycle of poverty and disease through the mechanism of increasing household income, and its effect has strengthened over time. Compared to other poverty reduction policies, major illness insurance has a greater influence on poverty alleviation.

## Introduction

Poverty has always been one of the major issues of concern in all nations worldwide. China's poor population was recorded as 16.6 million at the end of 2018, representing a significant decrease by Statistical bulletin of national economic and social development in 2018 issued by the National Bureau of Statistics; however, the data also indicate that more than 40% of families that had experienced poverty alleviation returned to poverty due to illness in 2018 according to National Health Commission. Among the poor in China, whether in urban or rural areas, a prominent reason for vulnerability to poverty is illness. Returns to poverty due to illness are the largest contributor to poverty in China. The proportion of households returning to poverty for this reason is more than 42% among poor households in China according to statistics from the National Health Commission of the People's Republic of China. Addressing such illness-induced returns to poverty is a major challenge of poverty reduction. In the long run, the government must focus more strongly on this issue.

China's National Medical Security Bureau, Ministry of Finance, and Poverty Alleviation Office of the State Council issued a 3-year action plan for poverty alleviation through medical security (2018–2020) in 2018, stating that by 2020, all rural poor would be covered by basic medical insurance, major illness insurance, and medical assistance. For major illness insurance, the plan noted that “we should increase the inclination of major illness insurance for the rural poor, reduce the starting line by 50%, increase the reimbursement ratio by 5%, and gradually increase and cancel the capping line.” Improving major illness insurance policies is considered essential to targeted poverty alleviation. By 2018, China's major illness insurance covered 1.129 billion urban and rural residents, and the actual reimbursement ratio for basic medical insurance increased by an average of 10–15% in 2019. The State Council's report proposed an increase in the standard per capita financial subsidy for resident medical insurance by 30 yuan, half of which would be used for major illness insurance. The council also proposed reducing and unifying the coverage threshold of major illness insurance and increasing the reimbursement ratio from 50 to 60% (1). Major illness insurance aims to offer “secondary reimbursement” in addition to basic medical reimbursement to improve the reimbursement proportion for medical treatment of serious illness. Major illness insurance is mostly calculated in sections and paid cumulatively. The “Notice on adequate basic medical security for urban and rural residents” of 2019 proposed that the proportion of reimbursement within the scope of major illness insurance policies be increased from 50 to 60% to further reduce patients' economic burden. The major illness insurance system has, in turn, increased its focus on the poor. Most provinces have policies in place to target major illness insurance to the poor in both urban and rural areas. Accordingly, the proportion of overall medical expenses borne by poor families declined to ~20% in 2017.

In the present environment integrating urban and rural medical insurance and popularization of major illness insurance in China, medical insurance—particularly of the latter type—plays a critical role in preventing and solving the problem of returns to poverty due to illness. The impacts of serious illness stretch many households' economic resources, result in budget constraints on household consumption, lead to household economic risks, and significantly increase the incidence of poverty. Major illness insurance, through the secondary reimbursement mechanism, can reduce patients' out-of-pocket medical expenses and improve residents' ability to afford catastrophic care-related expenses, thereby reducing the probability and severity of outsized household medical expenses (2) and decreasing the economic burden on patients suffering from major diseases. Facilitated by medical resource regulations, greater use of medical services by patients with major illnesses can allow them to recover and return to normal work, which, in turn, alleviates poverty resulting from major diseases. Major illness insurance can also reduce households' medical risk, promote consumption and investment, and improve income (3).

This research explores whether major illness insurance can reduce vulnerability to poverty by investigating the poverty reduction effect of major illness insurance, discussing the internal mechanisms of this effect and of returns to poverty due to illness, and ultimately suggesting policy directions to optimize poverty alleviation through major illness insurance. Furthermore, the research can help improve major illness insurance systems and implement effect poverty prevention. The research uses the random forest method to predict households' vulnerability to poverty. The standard poverty index statically measures families' welfare at a specific time point only; it does not consider future welfare or risks related to future welfare. As this index is an ex post measure that describes the state of poor groups at a certain time point, antipoverty policy formulated in accordance with it has limitations (4); however, poverty is a dynamic state. Research must consider prior analyses of poverty to conceive forward-looking poverty alleviation policies. A random forest algorithm is thus used to examine household characteristics, size, and composition, registered residence and region, and assets to construct a household poverty vulnerability index to predict the future risk of household exposure to the economic burden of major illness. An ex ante estimate of the impact and constraints on household survival and household members' development capabilities due to economic instability are derived. The resulting vulnerability to poverty index highlights the future possibility of household poverty, deepening the measurement of household poverty by means of a more comprehensive construct. For non-poor individuals, vulnerability refers to the risk of falling into poverty, and for poor individuals, vulnerability refers to the risk of becoming poorer. This index integrates static or comparative static analysis and dynamic analysis. The random forest method can reveal and analyze hidden characteristics and patterns in data using a classification algorithm trained on the sample data to identify and classify unknown data. Such a classification algorithm can be used in the assessment of big data to accurately identify poverty risks, transforming the basis of poverty identification from qualitative to quantitative criteria and moving targeting from a one-dimensional to a multidimensional approach. Comparing the degree of vulnerability to poverty among vulnerable households reveals differences across households, thereby helping to identify households with the most urgent need for poverty alleviation policies to ensure accurate specification of poverty alleviation objectives and improve the expected effect of social security policies.

The remainder of this paper is organized into five sections. Section 2 presents a literature review. Section 3 describes the data, variables, and measurements used in the study. Section 4 offers an overview of the results, and section 5 presents the robustness tests applied. Finally, section 6 concludes and elaborates on the policy implications.

## Literature Review

The World Development Report (WDR) 2000/1 emphasized the interaction of empowerment, security, opportunity, and poverty. This approach to the consideration of poverty introduced the concepts of risk and risk management as central to the policy dialogue on poverty reduction. Since this time, use of the term “vulnerability” has proliferated. It refers to the relationship of poverty, risk, and efforts to manage risk. A household's observed poverty level is *an ex* post measure of its well-being. Vulnerable families include all families whose welfare level is lower than the poverty line and families currently living above the poverty line but whose welfare status could deteriorate and leave them in poverty with the materialization of risk (5). The proportion of vulnerable families (or individuals) is higher than that of poor families (6). Bronfman (7) measured vulnerability to poverty in Chile by using survey panel data, determining that vulnerability to poverty affected more people than actual poverty. Anderloni et al. (8) assert that families' vulnerability to poverty derives from negative developments such as unemployment, reductions in working hours, death, disease, and other considerations. These sources of vulnerability to poverty are related mainly to their impact on household members' economic situations. Pritchett et al. (9) defined vulnerability as the probability of falling below the poverty line in any of the next three consecutive periods. Lighon and Schecter (10) consider the vulnerability poverty as extent of possible losses limited to a specific time period. Calvo and Deron (11) measure vulnerability as sensitive to loss. The increase of loss makes vulnerability increase at a faster and faster rate. Gunther and Harttgen (12) defined consumption shocks at both the household and community levels, indicating that impacts at the household level are the main cause of urban vulnerability, whereas rural vulnerability is related to impacts at the community level. Bourguignon and Goh (13) found unemployment to be the most important factor leading to vulnerability. Research frameworks developed in the literature on vulnerability to poverty include the theory of vulnerability to expected poverty (VEP) proposed by Pritchett et al. (14). Hoddinott and Quisumbing (15), Chaudhuri (16), and Klasen and Waibel (17) further developed VEP. Ligon and Schechter (10) presented the expected utility vulnerability (VEU) theory. Dercon and Krishnan (18) developed the risk exposure vulnerability (VER) theory. Using the utility-based insurance market value method based on expected expenditure and risk aversion, Finkelstein and McKnight (19) determined that medical insurance greatly reduces poverty (20), but the utility-based insurance value evaluation method is highly sensitive to the risk aversion parameter assumptions. There are no consistent findings in the literature on this topic (19), and the value of insurance may be underestimated for samples with incomes close to zero.

We apply a new method to measure vulnerability to poverty in this paper. The random forest algorithm is used to predict the vulnerability to poverty of family for the first time. This method is different from the expected poverty measurement method, which can reflect the different aspects of vulnerability, and has advantages over econometric methods and other machine learning methods.

The impact of major diseases on poverty vulnerability is mainly the result of two mechanisms: economic burdens and behavioral capacities. Regarding the former, both direct and indirect economic burdens of diseases are important considerations in the selection of disease treatment schemes. Although different indicators and methods may lead to different evaluations of economic burdens, major diseases increase such burdens on patients and can even lead to catastrophic poverty (21, 22). The occurrence of disease has a considerable impact on families and personal finances, especially those of low-income families, leading to household expenditures in excess of income and heavy debt. Even short-term serious disease may result in low-income families falling into long-term poverty (23). Moreover, as low-income families are sometimes forced to forgo necessary medical treatment, their quality of life and health status can further deteriorate, aggravating the probability of falling into poverty and entry into a vicious circle (4, 24). Russell (25) asserted that when people face illness, they have to pay medical expenses to fight diseases. When their expenditure reaches a certain degree, it can cause economic risks and descent into poverty. Pardhan and Prescott (26) elaborated the impact of disease shocks from two perspectives. First, medical expenses are incurred from disease to recovery. Second, disease reduces human capital and labor time, thus reducing income. Hoddinott and Quisumbing (15) found that every 10% decline in individuals' health level leads to a 6% increase in their vulnerability to poverty. Dercon and Hoddinott (27) found a significant positive relationship between serious illness and poverty; that is, poor families are more likely to suffer from the impact of serious diseases, and the degree of dispersion of economic risk from disease suffered by poor families is lower than that of non-poor families. Wagstaff (4) and Das et al. (24) found that the risk of major diseases leads to families with insufficient income falling into poverty. For low-income groups, the risk of major diseases and poverty influence one another. Low income makes families recoil when they face high medical expenses; they often choose to forgo treatment, which further worsens their health status and causes them to fall into a vicious cycle of poverty. Schneider (28) found diseases are an important cause of poverty that has a significant impact on families.

The purpose of medical insurance is to improve access to healthcare and potentially improve health (19, 29–32); however, a few studies have examined the potential poverty reduction effect of health insurance (33, 34). This research engages mainly with the aspects of reductions in the medical burden and catastrophic expenses and in risk reserve funds. The US Census Bureau (1979–2003) revealed that provision of public medical insurance may reduce the degree of household poverty as defined by supplementary poverty measures. Sommers and Oellerich (35) evaluated the poverty reduction effect of Medicaid, discovering that the Medicaid program reduced each beneficiary's out-of-pocket medical expenses from $871 to $376 and facilitated a reduction in the poverty rate among children by 1.0%, among disabled adults by 2.2%, and among elderly people by 0.7%. The Medicaid program in the United States was found to have lifted at least 2.6 million people out of poverty in 2010, with 3.4 million people lifted out of poverty overall, thereby making the program the third largest poverty alleviation plan in the United States. Huang (36) found that an urban housing security policy had an obvious poverty alleviation effect on low-income urban families and that this effect further increased over time through the impact of household education and training expenditure and labor force health. In contrast, some scholars have suggested that the poverty reduction effect of medical insurance is small or non-significant (37, 38).

The above review indicates that research on the poverty alleviation effect of medical insurance has not reached a unified conclusion. This paper attempts to answer four questions through further research. First, how can poor and vulnerable families be identified more accurately? Second, what is the effect of major illness insurance policies on poverty alleviation overall and among specific groups? Third, do serious illness policies buffer the well-being of vulnerable families who may become poor and return to poverty due to illness? Finally, is there a significant difference in the poverty alleviation effect of major illness insurance and group poverty alleviation policies?

## Data, Variables, And Poverty Vulnerability Measurement

### Data Source

China's major illness insurance system began in Zhanjiang, Guangdong Province in 2009. The six ministries and commissions of the State Council jointly issued guiding opinions for the delivery of major illness insurance for urban and rural residents, proposed the official establishment of such an insurance scheme, and began a nationwide pilot in 2012. In 2014, the State Council announced the acceleration of the serious illness medical insurance plan. The general office of the State Council proposed to fully implement the major illness insurance system for urban and rural residents throughout the country in 2015, and the program was in place by the end of 2015. The implementation was executed hierarchically, as is demonstrated below for the sample period. This data structure allows the rollout of major illness insurance to be regarded as a quasinatural experiment for the estimation of its effects.

The main data used in this paper are from the China Family Panel Studies (CFPS), an annual, nationally representative, longitudinal survey of Chinese communities, families, and individuals launched in 2010 with a focus on the economy and the well-being of the Chinese population. Topics covered include economic activities, educational outcomes, household dynamics and relationships, migration, and health. The CFPS sample covers 15,000 families in 635 villages in 162 counties of 25 provinces. The data set includes survey data from the base period of 2010 and follow-up data from 2012, 2014, and 2016. Among the sample provinces and cities, at the end of 2012, major illness insurance had been piloted in 1,468 counties, cities, and districts, while pilots for rural residents had been fully launched in the eight provinces of Liaoning, Jilin, Jiangxi, Henan, Hebei, Chongqing, Qinghai, and Ningxia. Most provinces and cities adopted the approach of overall municipal planning to gradually achieve a unified policy throughout the jurisdiction. For example, in 2013, Shizuishan City and Guyuan City conducted pilot work on major illness insurance according to the overall municipal planning model, launching the scheme in the entire region in 2014. Guangdong Province improved and promoted the “Zhanjiang model” in 2012. Based on the pilot projects in city of Shantou, Zhaoqing, Qingyuan and Yunfu, major illness insurance was officially implemented in more than 50% of prefecture-level cities in 2013, with the provinces fully implementing it in 2015. The base period of the study is 2010. In that year, only a few regions participated in the major illness insurance scheme. For 2012 and 2014, when the number of pilot cities gradually increased, the number of observations in our sample that participated in the major illness insurance program gradually increases. In line the policy rollout timeline, all our observations were participants in the major illness insurance plan in 2016. Individuals who were interviewed across each of the 2010, 2012, 2014, and 2016 waves were selected to compose the sample, excluding those with observed values of missing data, resulting in a final sample size of 30,630. The year 2010 is the base period prior to the implementation of the policy, 2012 and 2014 are the follow-up periods following policy implementation, and 2016 represents the period in which major illness insurance coverage had become comprehensive.

In order to study the impact of major illness insurance on the vulnerability to poverty of family samples who impacted by serious illness, we first need to judge whether a household is impacted by serious illness, then it is necessary to establish a measurable relationship between serious illness and the affordability of medical costs for a household. The standard definition of the impact of serious illness in China is based mainly on patients' cost level. When patients' annual out-of-pocket medical expenses exceed the minimum threshold to trigger major illness insurance, they are recognized as seriously ill patients. The guiding opinions on major illness insurance for urban and rural residents released in 2012 and 2015 specifies this threshold as an “annual cumulative burden of compliant medical expenses of individuals that exceeds the annual disposable income of urban residents and the annual per capita net income of rural residents in the previous year published by the local statistics department.” Academic works have also used differing definitions of an impact of serious illness. Gao and Yao (39) defined “serious illness impact” as “hospitalization (even for 1 day) or diseases with a total cost of more than 5,000 yuan.” Zhou et al. (40) defined catastrophic medical expenditure as a “proportion of self-paid hospitalization expenses in the total annual household expenditure that reaches 40%.” The WHO (41) defined household catastrophic expenditure as expenses incurred by families receiving medical and health services exceeding their actual ability to pay by 40%. In this study, referring to the standards of the WHO, we define a household as affected by serious illness with a dummy variable when the medical expenditure of the respondent's household in the current year exceeds 50% of the household income in that year. A numerical variable of the average gap in household catastrophic health expenditure is also constructed. Calculated based on the extent to which household health expenditure exceeds 50% of the threshold of catastrophic health expenditure, this index is used to reflect the severity of impact of serious diseases.

### Random Forest Algorithm

The random forest algorithm is an integration technology that introduces randomness into a variable set used for splitting at the node level, uses the bootstrap resampling method to extract multiple samples from the original sample, conducts decision tree modeling for each bootstrap sample, and establishes a relationship between several input and outcome variables by generating a large number of classification trees. Through K rounds of training and combination of the training of multiple decision trees, a function that connects the characteristics of vulnerability to poverty with poverty outcomes is generated, the function's performance is evaluated, and its out-of-sample prediction performance is tested. Based on learning of the relationship between the characteristics of vulnerability to poverty and poverty tag values, an optimal model is constructed (42) and used to output the classification probability (the mean of the output probability of all decision trees), which represents vulnerability to poverty.

The random forest prediction of vulnerability to poverty is the unweighted average of the set:


(1) $$\begin{array}{l} h\left(x;\theta_{k}\right)=\left(\frac{1}{K}\right)\overset{K}{\underset{k=1}{\sum }}h\left(x;\theta_{k}\right) \end{array}$$

Equation (1) shows that multiple voting methods are used to determine the final poverty classification results, where *k* = 1, …, *K*, K represents a group of identically distributed but related regression trees, θ_*k*_ represents the *k*^*th*^ decision tree, and X represents the input vector and random vector X of length P. The input vector is a variable reflecting the characteristics of vulnerability to poverty, comprised of 54 variables, including the main variables shown in Table 1, θ_*k*_ is an independent and equally distributed random vector that determines the growth process of a single course decision tree, *h*(*x*; θ_*k*_) is a single decision tree classifier, and $\overset{K}{\underset{k=1}{\sum }}h\left(x;\theta_{k}\right)$ represents a set of decision trees generated by the random forest training algorithm. Y is the result variable in the model. It classifies whether a household is poor and is expressed by 1 (poor) or 0 (not poor). The training data are independent of the joint distribution of (x, y), which is determined by n(p+1)(x_1_, *y*_1_), …, (*x*_*n*_, *y*_*n*_) and consists of n(p+1) groups. Therefore, the value of vulnerability to poverty of the random forest output is equal to the mean probability of vulnerability to poverty of all decision trees.

**Table 1:** Indicators of vulnerability to poverty.

**Index**	**Meaning**	**Index description**
**Household head characteristic variables**		
Gen	Gender	1 for male; 0 for female
Age	Age	Age of head of household (years)
Mar	Marital status	1 for unmarried; 2 for married (with spouse); 3 for divorced; 4 for widowed
Res	Residence type	0 for rural residents; 1 for urban residents
Edu	Education	Education level, divided into four groups: primary school and below, junior middle school, senior high school and junior college and above. Primary school and below as the benchmark group, 0 for primary school and below, 1 for junior middle school, 2 for senior high school, and 3 for junior college and above
Emp	Employment status	Unemployment, employment and withdrawal from the labor market, with unemployment as the benchmark group, 0 for unemployment, 1 for employment, 2 for withdrawal from the labor market
Job	Type of job	0 for government departments/party and government organs/people's organizations and institutions; 1 for state-owned enterprises, foreign businessmen/Hong Kong, Macao and Taiwan enterprises, other enterprises; 2 for private enterprises/individual industrial and commercial households, individuals/families; 3 for private nonenterprise organizations/associations/guilds/foundations/village or neighborhood committees; 4 for others
Heas	Short-term health level	Degree of disease and injury. 0 for none, 1 for not serious, 2 for average, 3 for serious
Heal	Long-term health level	1 for chronic disease, 0 for no chronic disease
**Household characteristics**		
Hou	House property	Total market price of current residential housing
Fas	Household size	Total number of families
Tas	Household net assets	Total household assets minus household liabilities, taken as logarithm
Chi	Number of minor children	Number of children under 18 in the household
Soc	Social network	Social network support, represented by expenditure on human gifts^1^
Inc	Per capita household income	Household income includes operating income, wage income and property income, taken as logarithm
Med	Medical expenditure	Total medical expenditure of the household, not including expenses that have been reimbursed and are expected to be reimbursed, but including portions lent or paid by relatives and friends^2^
**Regional characteristics**		
Eco	Regional economic development level	Per capita GDP of the city where the household is located (10,000 yuan)
Dis	Natural disasters	Direct losses from geological disasters in this area (10,000 yuan)

When *k* → ∞, the law of large numbers ensures that:


(2) $$\begin{array}{l} E_{X,Y}{\left(Y-\bar{h}h\left(X\right)\right)}^{2}\to E_{X,Y}{\left(Y-E_{\theta }h\left(X;\theta \right)\right)}^{2} \end{array}$$

The right side of Equation (2) represents the error of the random forest prediction of vulnerability to poverty, which can be written as $PE_{f}^{*}$. Convergence of this formula means that the random forest will not be overfitted.

The average prediction error H (x) of vulnerability to poverty of a single decision tree θ is:


(3) $$\begin{array}{l} {PE}_{t}^{*}={E_{\theta }E}_{X,Y}{\left(Y-h\left(X;\theta \right)\right)}^{2} \end{array}$$

Suppose that for all θ, the decision tree is unbiased; that is, *EY* = *E*_*X*_*h*(*X*; θ), such that:


(4) $$\begin{array}{l} {PE}_{f}^{*}\leq \bar{\rho }E_{t}^{*} \end{array}$$

where $\bar{\rho }$ is the residual *Y*−*h*(*X*; θ) and θ and *Y*−*h*(*X*; θ^,^) are weighted correlations. Equation (4) presents the conditions required by the random forest method to predict vulnerability to poverty. The low correlation between the residuals of different trees in the random forest, the prediction error of a single tree, and the random forest passing factor $\bar{\rho }$ reduce a single tree error to $\bar{\rho }E_{t}^{*}$.

### Variables

#### Major Illness Insurance

The key explanatory variable is major illness insurance. The answer to the question “What kind of medical insurance coverage does the household have?” in the adult questionnaire is matched with the observation year and province to identify whether major illness insurance was available. Since there is no information regarding major illness insurance in this survey, we refer to the implementation process of this insurance scheme, which was rolled out for urban and rural residents from 2012, with full coverage achieved in 2015. Therefore, 2010 is regarded as the reimplementation period of the policy, 2012 and 2014 are the policy implementation period, for which we match the timing of implementation with the provinces and cities where different families are located, and 2016 is considered the period of full policy implementation.

#### Vulnerability to Poverty

Vulnerability to poverty is an ex ante estimate of the constraints on household survival and household members' development ability caused by household exposure to future risks, shocks, and vulnerability to economic instability, which depends on the household's future monetary welfare, human capital, health, infrastructure, and public services and the degrees of change in these aspects. The reason for vulnerability to poverty is exposure to synergistic or heterogeneous risk. When families face a relatively high level of potential risk or risk exposure and their ability to cope with risk is limited, their vulnerability to poverty is higher. Household vulnerability to poverty is an ex ante indicator of household welfare that helps to analyze which non-poor households may fall into poverty in the future or which families that have been lifted out of poverty may fall into poverty and become poor again in the future. The difference between vulnerability to poverty and poverty is also inherent to the existence of risks. If families do not face risks, the state of household vulnerability to poverty and welfare risk management will be relatively stable over a certain period of time, non-poor households will not fall into poverty, and families that have been lifted out of poverty will not return to poverty.

If the fixed effect of the outcome variable poverty is considered, the model should contain a large number of explanatory variables, but when the general linear regression model contains too many variables, the estimation results will be biased, and many variables are multicollinear, which makes the prediction ability of the model outside the sample very poor. As described, the random forest model can handle the situation with a large number of explanatory variables, and has the ability to screen independent variables, which significantly improves the accuracy of prediction (43), so the random forest algorithm is used to analyze vulnerability to poverty at the household level in this research. We use the random forest algorithm to measure the vulnerability to poverty by examining the sources of vulnerability by characterizing risks and shocks faced by the population We select the relative poverty standard as the result variable. Relative poverty generally refers to the lack of material data and low consumption ability compared with others. Due to the differences between urban and rural household, 70% of the per capita net income of urban and rural samples in different years are used, respectively (44) as the relative poverty line of income.

Referring to the existing literature, the vulnerability to poverty depends on the future income prospect, the degree of income fluctuation faced, and its ability to consume stably in the face of income or other livelihood shocks, which in turn depends on the complex dynamic relationship between the environment (macroeconomic, institutional, socio political and material environment), in which the family operates and the resources, manpower material and financial resources and their behavioral responses. Based on this, we select the input variables according to the impact faced by the family, available resources, family burden and family characteristics.

Considering the possible impact on families as the root cause of the vulnerability to family poverty, as the World Bank's survey on the global poor found that the inability to cope with the impact is the main cause of poverty (45). Anderloni et al. (8) believe that the poverty vulnerability of families comes from vulnerable negative impacts, such as unemployment, reduction of working hours, death of family members, major diseases, etc. Bourguignon et al. (46) found that unemployment is the most important factor leading to vulnerability. The root causes of poverty may be family specific, such as disease, fire and local unemployment, or the whole community or specific regions, such as natural disasters and epidemic situations. In 2020, a total of 22.489 million people were affected by various natural disasters in China, with a direct economic loss of 65.78 billion yuan. It may also be macroeconomic, such as the financial crisis economic recession and other economic shocks leading to unemployment or inflation (12). We select input variables such as medical expenditure, employment status and major events from the aspects of special shocks and synergistic shocks.

In the face of impact, some families will fall into poverty, while some families can pull through easily. Therefore, the ability of families to cope with risks will affect the vulnerability to poverty of families. The resources of families to cope with risks need to be considered (47), if families lack coping mechanisms such as insurance and access to credit, people may easily fall into poverty. Family social network can directly affect the poverty vulnerability of families, or reduce the poverty vulnerability by helping families obtain external resources. Therefore, input variables such as real estate, family assets, social network and family income are selected, Among them, the indicators of family assets include “bank loans other than housing loans,” “operating assets,” “value of durable consumer goods,” “total family financial assets,” “productive fixed assets,” “total family real estate,” “land assets” and other indicators, and the indicators reflecting income include “total family income,” “net family income,” “wage income” and “property income” Wait.

Family characteristics reflect the family situation and the family's ability to bear risks to a great extent. Input variables such as family size, number of minor children, demographic characteristics of head of household and family characteristics are selected.

Our random forest data set ultimately contains a total of 48 variables, including the main variables presented in Table 1. From the perspective of personal characteristics, indicators such as gender, age, marital status, employment, type of work, and health status are important. Among the household characteristics, housing, household size, household net assets, the number of minor children, social network, household per capita income, and medical expenditure are important. Regional characteristics include the per capita economic growth level and natural disasters in the city where the household is located. Real estate, household net assets, household per capita income, regional GDP, and direct economic losses from natural disasters are also included in logarithmic form.

#### Other Variables

Demographic, economic, and social characteristics of the head of household, household characteristics and urban characteristics are also included, for a total of 50 indicators across three categories. The corresponding characteristics for the head of household are age, gender, education, and employment status. Household characteristics reflect the household situation and the extent to which the household can bear risk, captured through the aspects of income, household size, social capital, and other relevant indicators. The third category reflects the characteristics of the region to which the observation belongs. China is an enormous country with vastly different levels of economic development and susceptibility to natural disasters in different regions. These differences may affect the dispersion of risk resilience among families, so it is necessary to consider the corresponding variables.

### Measurement of Vulnerability to Poverty

**Figure 1:**
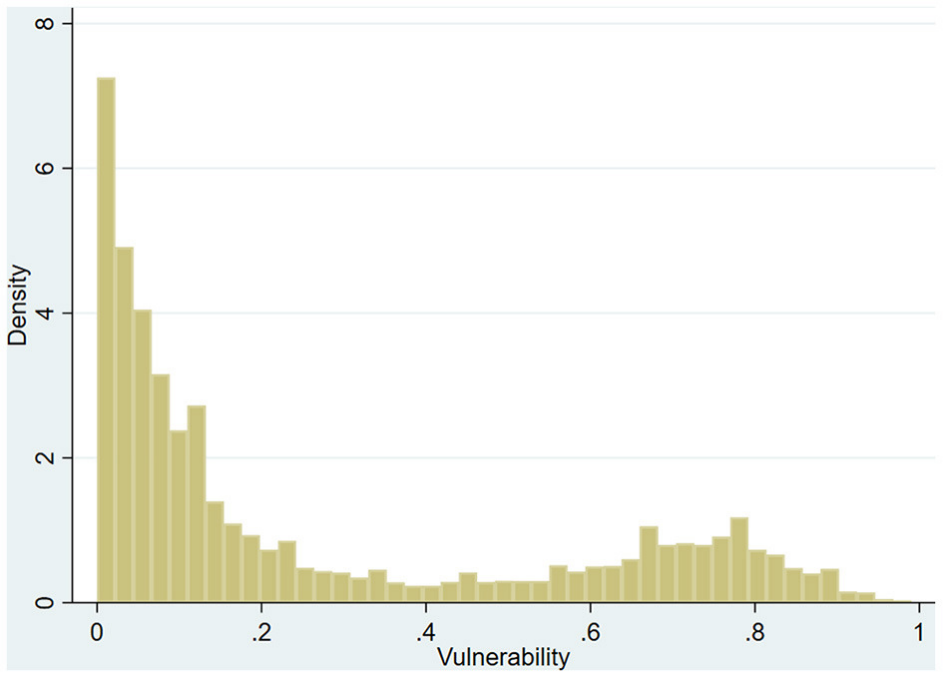
Histogram of vulnerability to poverty.

By selecting some observations as the test set and some as the training set, we achieve model prediction accuracy of 95%. According to the prediction results of the model, the vulnerability to poverty of each household is calculated. The frequency analysis results are presented in Figure 1.

As seen in Figure 1, the average vulnerability to poverty is 10%, with a right-skewed distribution, and skewness of the vulnerability to poverty of sample household is 0.903.

### Research Model

#### Fixed Utility Model

Considering the issue of missing variables and errors, this research applies the fixed effect model to improve the consistency of the estimation results. The fixed utility model is as follows:


(5) $$\begin{array}{l} {Pov}_{it}=\alpha_{i}+\beta_{1}{Ins}_{it}+\varepsilon_{it} \end{array}$$

where the explanatory variable is *Pov*_*it*_, the vulnerability to poverty variable, which incorporates a series of indicators affecting household vulnerability to poverty, and *Ins*_*it*_ indicates major illness insurance.

#### Difference in Differences Model Based on Propensity Score Matching

To more accurately estimate the policy effect, we matched the treatment and control groups in the base period. The propensity score matching method was used to match various explanatory variables in the base period experimental and control groups, control for heterogeneity in the demographic, economic, and household characteristics of the two groups, and generate a matched data set. There are differences between the families participating in major illness insurance before and after the scheme's implementation, and at the same time, there are differences between families covered and families not covered by major illness insurance. Families with coverage are regarded as the treatment group, and those not covered are taken as the control group. Using the difference in differences (DID) model, we compare the treatment and control groups to assess the impact of major illness insurance on household vulnerability to poverty. The main purpose of this method is to obtain an effect similar to that of the experiment and achieve a similar counterfactual through estimation of the double difference in the absence of the experiment to obtain the net effect, which is average treatment effect for the treated group (ATT) of major illness insurance on poor and vulnerable families. The following double difference model is constructed:


(6) $$\begin{array}{l} {Pov}_{it}=\alpha_{i}+\beta_{1}{Ins}_{it}+\beta_{2}{Year}_{it}+\beta_{3}{Year}_{it}*{Ins}_{it}+\varepsilon_{it} \end{array}$$

where *Pov*_*it*_ represents families' vulnerability to poverty, *Ins*_*it*_ indicates whether the household has major illness insurance, *d* = 1 is the treatment group and *d* = 0 is the control group, time indicates the period during the rollout of major illness insurance, 0 indicates that the individual did not participate in major illness insurance in $Year_{it}^{*}$, and 1 indicates that the individual participated in major illness insurance in *Year*_it_*. *Ins*_*it*_ represents the ATT of major illness insurance on vulnerability to poverty, that is, the average treatment effect on the experimental group, which is the net impact of major illness insurance on vulnerability to poverty after other effects are excluded, and β_3_ is the double difference statistic.

## Results

### Descriptive Statistics

Table 2 presents the mean, standard deviation, minimum, and maximum values of the total sample and the sample families impacted by serious disease in the base year. Little difference is seen in the mean values of most variables for the sample of households impacted by serious illness and the total sample.

**Table 2:** Summary statistics.

**Variable**			**Total sample**			**Serious illness sample**		
	**Mean value**	**Standard deviation**	**Minimum**	**Maximum**	**Mean**	**Minimum**	**Maximum**	**Mean**
Gen	0.585	0.493	0	1	0.61	0	1	0.489
Age	50.647	13.572	16	95	58.73	17	94	13.799
Mar	2.129	0.578	1	4	2.26	1	4	0.725
Res	0.301	0.458	0	1	0.27	0	1	0.442
Edu	0.771	0.939	0	3	0.46	0	3	0.752
Emp	1.228	0.510	0	2	1.41	0	2	0.579
Job	0.848	0.936	0	3	0.7	0	3	0.811
Heas	1.206	0.358	0	3	1.547	0	3	0.462
Heal	0.147	0.358	−8	1	0.31	−2	1	0.464
Hou	6.776	2.740	0	13.922	5.7641	0	12.62	2.917
Fas	3.757	1.796	1	26	3.33	1	14	1.87
Tas	11.866	2.013	0	17.835	11.183	0	16.06	2.210
Chi	1.957	1.180	0	10	2.41	0	10	1.375
Soc	7.445	1.275	0	12.766	6.896	0	10.821	1.320
Inc	10.056	1.317	0	16.156	9.4629	1.613	12.822	1.403
Eco	9.923	0.717	7.049	11.300	9.909	8.166	11.323	0.729
Dis	8.509	2.221	−0.693	12.368	8.444	−0.698	12.372	2.240
*N*			30,630			30,630		

### Major Illness Insurance and Poverty Vulnerability

#### Baseline Estimates

The fixed effect model is used to test the impact of major illness insurance on vulnerability to poverty. The regression results are presented in Table 3.

**Table 3:** Fixed utility model test results of the impacts of major illness insurance on poverty vulnerability.

	**Interpreted variables: vulnerability to poverty**			
**Variable**	**Total sample**	**Households impacted by severe illness**	**Households impacted by severe illness (average gap ≥ 50)**	**Households impacted by severe illness (average difference <50)**
Cons	13.379*** (1.246)	9.998*** (1.323)	13.848*** (1.467)	5.283* (2.842)
*Ins*_*it*_	−0.334* (0.735)	−0.339*** (0.0007)	−0.342*** (0.0007)	−0.336*** (0.0008)
Year fixed effects	Included	Included	Included	Included
Province fixed effects	Included	Included	Included	Included

***, * indicate significance levels at 1 and 10%, respectively.

#### PSM-DID Test

Selecting different control variables and making matching adjustments can reduce selection bias and confounding bias caused by self-selection problems to a large extent sample; we use this procedure to form a three-phase balanced panel for 2010–2012 (456 households), 2010–2014 (490 households), and 2010–2016 (544 households).

The results of the DID test in Table 4 indicate a significant effect of major illness insurance in alleviating household vulnerability to poverty at the 1% confidence level. Over time, the effect of major illness insurance on vulnerability to poverty has gradually increased. In 2012, major illness insurance reduced vulnerability to poverty by 30.7%, in 2014, by 31.9%, and in 2016, by 36%. A possible reason for this strengthening effect is that the major illness insurance scheme is comprehensive and the reimbursement proportion has also increased, possibly strengthening its role in hedging the economic risk of disease. The cross term of time and major illness insurance reflects the net effect of major illness insurance on vulnerability to poverty. The panel data demonstrate that major illness insurance did indeed significantly reduce vulnerability to poverty by 20.7%, 21.2%, and 25.2% across the three phases.

**Table 4:** Difference in differences (PSM-DID) test.

	**Dependent variable: vulnerability to poverty**		
**Variable**	**PSM-DID** **2010–2016**	**PSM-DID** **2012–2016**	**PSM-DID** **2014–2016**
Cons	0.122*** (0.031)	0.095*** (0.031)	0.097*** (0.031)
*Ins*_*it*_	−0.307*** (0.051)	−0.319*** (0.042)	−0.360** (0.048)
*Year*_*it*_	0.002* (0.053)	0.003* (0.052)	0.002* (0.055)
*Year*_it_*Ins_*it*_	0.207** (0.076)	0.212** (0.059)	0.252** (0.063)

***, **, and * indicate significance levels at 1, 5, and 10%, respectively.

#### Effects of Poverty Reduction Under Different Threshold, Upper Limit and Reimbursement Proportion

In practice, provinces and cities in China have formulated policies according to local conditions based on the basic principle of fiscal responsibility. The threshold for triggering coverage and the upper limit of reimbursement in the major illness insurance scheme are different in provinces and cities in China affected by differing economic conditions, per capita income, and financing levels of basic medical insurance, Differences also exist in the overall planning level and the definition of serious illness. If we take Beijing as an example, the starting standard of major illness insurance in 2019 was 15,202 yuan, and the payment limit was 25,000 yuan. The threshold for the reimbursement proportion was 65% for 50,000 yuan and 75% for more than 50,000 yuan. Only out-of-pocket household medical spending is collected in the questionnaire, and the reimbursement proportion is calculated according to the out-of-pocket amount. Since the questionnaire does not investigate the starting threshold and payment limit, we match these data to those of the annual major illness insurance policy in the area where each household is located.

Table 5 demonstrates that reducing the starting threshold at which major illness insurance is triggered and increasing the payment limit and reimbursement ratio could reduce the poverty vulnerability of the sample families.

**Table 5:** Effects on poverty reduction.

**Variable**	**Dependent variable: Vulnerability to poverty**		
Cons	0.489*** (0.070)	0.253*** (0.062)	0.266*** (0.062)
Deductible	0.072** (0.048)		
Payment limit		−0.297*** (0.108)	
Reimbursement ratio			−0.205*** (0.057)
Year FE	Included	Included	Included
Province FE	Included	Included	Included

***, ** indicate significance levels at 1 and 5%, respectively.

### Subsample Test

The subsample of rural households is chosen to test the target of poverty alleviation. Table 6 demonstrates that major illness insurance has a significant impact on vulnerability to poverty among rural families, and this effect is greater than that in the total sample. Major illness insurance alleviates vulnerability to poverty in the overall rural sample by 40.4%, alleviates impacts of serious illness on rural families by 42.9%, and alleviates severe impacts of serious illness on rural families by 44.4%. Alleviation of lesser impacts on rural families of serious diseases is relatively small, and the effect of the overall sample is 37.7%.

**Table 6:** Test results of the effect of major illness insurance on vulnerability to poverty among rural residents.

	**Dependent variable: vulnerability to poverty**			
**Variable**	**Total rural sample**	**Sample of families affected by serious diseases in rural areas**	**Sample of families affected by serious diseases in rural areas (average difference greater than or equal to 50%)**	**Sample of families affected by serious diseases in rural areas (average difference is greater than 50%)**
Cons	0.084*** (0.003)	0.790 (0.003)	0.780 (0.005)	0.793 (0.003)
*Ins*_*it*_	−0.404* (0.063)	−0.429*** (0.007)	−0.444*** (0.027)	−0.377*** (0.013)
Year FE	Included	Included	Included	Included
Province FE	Included	Included	Included	Included

***, * indicate significance levels at 1 and 10%, respectively.

## Robustness Tests

### Lagged Phase Test

Considering that there may be a certain time lag in the role of the major illness insurance scheme, and to avoid endogeneity problems, this paper tests the variable measuring household vulnerability to poverty by using the fixed utility model with lagged values.

As presented in Table 7, the effect of major illness insurance on vulnerability to poverty in the overall sample is 34.7%, the effect for families affected by serious illness is 35.8%, that for families severely affected by serious illness is 36.1%, and that for families affected to a lower degree by serious illness is 34.9%. If we compare these results with those of the first model, the role of major illness insurance in alleviating poverty is increased to a certain extent, indicating a certain time lag in the effect of the major illness insurance rollout.

**Table 7:** Major illness insurance and vulnerability to poverty Lag phase I.

	**Dependent variable: vulnerability to poverty**			
**Variable**	**Total rural sample**	**Sample of families affected by serious diseases**	**Sample of families affected by serious diseases (average difference greater than or equal to 50%)**	**Sample of families affected by serious diseases (average difference is greater than 50%)**
Cons	13.338*** (1.487)	9.935*** (1.478)	13.356*** (1.293)	5.746* (2.487)
*Ins*_*i*(*t*−1)_	−0.347* (0.647)	−0.358*** (0.0007)	−0.361*** (0.0007)	−0.349*** (0.0008)
Year FE	Included	Included	Included	Included
Province FE	Included	Included	Included	Included

***, * indicate significance levels at 1 and 10%, respectively.

### Major Illness Insurance and the Vicious Circle of Poverty and Disease

Amartya (48) asserted that there are two main factors leading to poverty. The first is that individuals lack necessary capabilities for survival and development and thus lose access to channels for accessing income sources. The second is that individuals are deprived of the right to acquire these capabilities. Diseases deprive individuals of their capabilities, resulting in the loss of income sources and poverty. In terms of behavioral capabilities, major diseases cause patients to lose their working ability, and access to income sources can be blocked, which increases the probability of families falling into poverty and of poor patients falling into the vicious circle of poverty and disease.

Major illness insurance can enhance and stabilize income (38). In regard to the enhancement effect, improvements in labor force health can increase income through higher labor efficiency and a larger labor supply. In terms of the stability effect, improvements to the health status of the labor force not only make up for labor time lost to diseases but also reduce the medical expenses caused by physical diseases in the long run, reduce the uncertainty of impacts on future income, and promote families' ability to expand their human and material capital investments to increase their income level (49).

Table 8 indicates that the model coefficient of income as an intermediary mechanism is significant, making *Inc*_*it*_ a path variable of *Ins*_*it*_, and due to absorption of the original effect by the path variables, the coefficient of major illness insurance decreases in comparison with that in model (5). Therefore, major illness insurance affects vulnerability to poverty through path variables. Major illness insurance can both enhance and stabilize income, thus further alleviating the vicious circle of poverty and disease.

**Table 8:** Test of additional income as an intermediary mechanism.

**Variable**	**Dependent variable: vulnerability to**			
	**poverty (total sample)**			
	**Coefficient**	**Standard error**	**LLCI**	**ULCI**
Cons	0.442***	0.034	0.376	0.508
*Ins*_*it*_	−0.234***	0.003	−0.041	−0.028
*Inc*_*it*_	−0.31***	0.036	−0.08	0.059
*Ins*_it_*Inc_*it*_	0.051***	0.004	−0.006	0.008

*** indicate significance levels at 1%, respectively.

The mechanism whereby disease affects income and poverty is analyzed, with intermediary variables added on the basis of the original model (1) to establish an intermediary effect model:


(7) $$\begin{array}{l} {Inc}_{it}=\alpha_{i}+\beta_{1}{Ins}_{it}+\beta_{2}{Inc}_{it}+\beta_{3}{Ins}_{it}*{Inc}_{it}+\varepsilon_{it} \end{array}$$

where *Inc*_*it*_ is a path variable. Equation (7) tests whether major illness insurance has an indirect impact on vulnerability to poverty through *Inc*_*it*_.

### Comparison Between Major Illness Insurance and Other Poverty Reduction Systems

#### Public Transfer

Public transfer payments are a critical poverty reduction policy. These payments are a government expenditure that is not compensated with labor services or commodities. Agostini and Brown (50) found that cash subsidies have a significant role in reducing poverty and inequality. Jha (51) found that two public policies, work subsidies and food subsidies, significantly alleviated poverty in India. This paper compares the effects of major illness insurance and other poverty reduction measures. The CFPS database includes the amount of public transfer payments. In this paper, two indicators of receipt of public transfer payments are selected, namely, whether families received government subsidies and whether they received social donations, as presented in Table 9.

**Table 9:** The poverty reduction effect of major illness insurance.

	**Dependent variable: vulnerability**		
	**to poverty (total sample)**		
**Variable**	**Major illness**	**Government**	**Social**
	**insurance**	**grants**	**contributions**
Cons	13.379*** (1.246)	0.090*** (0.002)	0.090*** (0.002)
*Pov*_*it*_	−0.334* (0.735)	−0.018*** (0.003)	−0.039*** (0.003)
Year FE	Included	Included	Included
Province FE	Included	Included	Included

***, * indicate significance levels at 1 and 10%, respectively.

The results indicate that government subsidies and social donations affect vulnerability to poverty at a confidence level of 1%, but the impact is small, reducing vulnerability to poverty by 1.8 and 3.9%, respectively; these results are slightly different from those found by Fan and Jie (52). There are four possible reasons for this. First, the data sets used in this paper and in Fan and Jie are different. Second, the method of calculating vulnerability to poverty in this paper differs from Fan and Jie's approach. Third, the variables selected in this paper differ from those selected by Fan and Jie. Fan and Jie's research takes cash income from hardship subsidies, disability subsidies, or welfare funds as proxies for public transfer payments, whereas the two public transfer payment variables selected in this paper capture whether the household receives government subsidies or a pension. Fourth, Fan and Jie selected data from 2006 and 2009, whereas this paper uses data from 2010 to 2016. With the change in the sample period, the impact of transfer payments on vulnerability to poverty may also be evolving.

#### Infrastructure Construction

Consolidation and expansion of poverty alleviation depends on infrastructure investment and asset empowerment under the guidance of the government (53). Among these, infrastructure construction is extremely important for achieving sustainable development. This research uses variables regarding infrastructure construction from the CFPS database in an OLS model to test the relationship between infrastructure construction and vulnerability to poverty.

There is a negative correlation between infrastructure construction and vulnerability to poverty in Table 10, which shows that for every 1% increase in infrastructure construction, the family poverty vulnerability is significantly reduced by 2.0 percentage points. It can be seen that the improvement of infrastructure can effectively reduce the poverty vulnerability, Infrastructure construction should be regarded as the basic condition for poverty-stricken areas to completely get rid of poverty and an important guarantee for poverty-stricken areas to get rid of poverty and never return to poverty.

**Table 10:** Test results of the effect of infrastructure construction on vulnerability to poverty.

**Variable**	**Dependent variable: vulnerability**
	**to poverty (total sample)**
	**Relative poverty label**
Cons	2.186*** (0.045)
Infrastructure construction	−0.020*** (0.004)
Control variables	Included
Province FE	Included
*R* ^2^	0.741
Adjusted *R*^2^	0.740
N	20,936

*** indicate significance levels at 1%, respectively.

#### Investment in Education

China's poverty alleviation policies emphasize the important mission of education in combining poverty alleviation with support for intelligence and ambition, continuously strengthening cultural and technical education and knowledge for the poor, enhancing their self-development ability, and preventing intergenerational transmission of poverty. General economic theory holds that education can benefit families through direct and spillover effects. Good education improves individuals' technical knowledge and can increase household income. The household education investment variables in the CFPS database are selected for use in an OLS model to test the relationship between household education investment and vulnerability to poverty. Due to the lag in returns to education investment, we also apply lagged household education investment as an independent variable to investigate the long-term effect of this variable.

Table 11 shows that there is a negative correlation between family education expenditure and poverty vulnerability. For every 1% increase in family education expenditure in the current period, the family poverty vulnerability is significantly reduced by 0.5042 percentage points, the increase in family education expenditure in the previous period significantly reduces the family poverty vulnerability by 0.5149 percentage points, and the increase in family education expenditure in the previous two periods significantly reduces the family poverty vulnerability by 0.4049 percentage points, The increase of family education expenditure in the last three periods significantly reduced family poverty vulnerability by 0.1564 percentage points, which is the same as Si (54) conclusion that family education expenditure significantly reduced farmers' poverty vulnerability. It can be seen that family education expenditure continues to have a significant impact on poverty vulnerability.

**Table 11:** Test results of the effect of education investment on vulnerability to poverty.

**Variable**	**Dependent variable: vulnerability to poverty (total sample)**			
Cons	1.182*** (0.153)	1.088*** (0.128)	0.925*** (0.156)	1.309*** (0.333)
Investment in Education	−0.005042*** (0.0009)			
Investment in education in the previous period		−0.005149*** (0.001)		
Investment in education in the last two periods			−0.004049*** (0.002)	
Investment in education in the last three periods				−0.001564*** (0.003)
Control variables	Included	Included	Included	Included
Province FE	Included	Included	Included	Included
*R* ^2^	0.599	0.605	0.606	0.638
Adjusted *R*^2^	0.598	0.602	0.602	0.618
N	13,365	6,619	4,033	840

*** indicate significance levels at 1%, respectively.

## Conclusions and Policy Recommendations

This paper investigates the impact of major illness insurance on household vulnerability to poverty. The random forest method is applied to measure vulnerability, followed by fixed effect and PSM-DID models to correct selection bias and test the effect of major illness insurance. In addition, the vicious circle of disease and poverty and a comparison of major illness insurance with other poverty reduction measures are examined in the robustness analysis. In general, the research finds that major illness insurance has an obvious role in targeted poverty alleviation, and this role increases with the degree of protection offered by the insurance. Compared with government subsidies and social donations, major illness insurance appears to alleviate poverty more effectively. At the same time, other poverty alleviation measures should also be considered to increase the alternative value of household resources. Alternative value may underestimate the value of major illness insurance for poor and vulnerable families.

In view of the above, corresponding major illness insurance policies should be formulated based on the determinants of vulnerability to poverty to achieve targeted poverty alleviation. First, to achieve an accurate mechanism for identifying poverty, the random forest method was applied to estimate vulnerability to poverty, finding household income and social networks to be key factors affecting vulnerability. Application of the random forest method, compared with traditional poverty identification methods, has more advantages for assessing interactivity, non-linearity, and heterogeneity.

Second, the major illness insurance scheme currently operates based on medical expenses; while this approach can achieve universal coverage, it still comes up short in terms of accurate targeting. Although major illness insurance in some areas is focused on the poor, especially individuals with filing poverty cards and low-income, severely disabled and other poor people, its scope of application remains limited. A certain gap exists in achieving the policy goal of reducing catastrophic health expenditures among low-income families. The risk of high medical expenses of seriously ill patients cannot be effectively resolved, and so the phenomenon of poverty and returns to poverty due to illness cannot be completely avoided. Scientific methods must be adopted to optimize the standards of major illness insurance. Focusing on the circumstances of major diseases and rare diseases among the economically poor can improve the accuracy of targeting in major illness insurance protection.

Finally, the effect on vulnerability to poverty was considered to facilitate the setting of more accurate thresholds, encourage the establishment of a dynamic adjustment mechanism linking individual payment and threshold standards with household income, and gradually improve the proportion of medical insurance reimbursement based on the degree of poverty to alleviate the burden of medical expenses among targeted poor households.

## Data Availability Statement

Publicly available datasets were analyzed in this study. This data can be found here: http://www.isss.pku.edu.cn/cfps/.

## Author Contributions

LZ: writing—conceptualization, methodology, original draft, and formal analysis. LP: writing—review and editing. Both authors contributed to the article and approved the submitted version.

## Funding

We gratefully acknowledge the financial support from the National Natural Science Foundation of China (No. 71903209).

## Conflict of Interest

The authors declare that the research was conducted in the absence of any commercial or financial relationships that could be construed as a potential conflict of interest.

## Publisher's Note

All claims expressed in this article are solely those of the authors and do not necessarily represent those of their affiliated organizations, or those of the publisher, the editors and the reviewers. Any product that may be evaluated in this article, or claim that may be made by its manufacturer, is not guaranteed or endorsed by the publisher.

## Footnotes

^1^Major events include weddings and funerals, children's birth, children's school, which can reflect the family's consumption ability and family's social communication ability when facing the impact of major events.

^2^The CFPS questionnaire is slightly adjusted every year. In regard to the calculation of medical expenditure, the CFPS did not separately ask the family about its out-of-pocket medical expenditure in 2010. The question was “What was the family health care expenditure in the past year?”, and so we use family health care expenditure instead of family out-of-pocket medical expenditure. From 2012, the survey began to include the item “What was the medical expenditure directly paid by your family in the past year?”
